# The Ubiquitination of *Mycobacterium tuberculosis* Rv3717 Promotes Proteasomal Degradation of Interleukin Enhancer-Binding Factor

**DOI:** 10.3390/biology14101414

**Published:** 2025-10-14

**Authors:** Xu-Wen Gui, Teng-Fei Zhang, An-Qi Zheng, Ming-Xin Guo, Qian-Wei Dong, Tao Jiang

**Affiliations:** 1 Department of Biotechnology, College of Basic Medical Science, Dalian Medical University, Dalian 116044, China; guixvwenn@163.com (X.-W.G.); ztf2640007013@163.com (T.-F.Z.); qaz1739122215@163.com (A.-Q.Z.); gmx1440691021@163.com (M.-X.G.); 2 Department of General Practice, Lujiazui Community Health Service Center, Shanghai 200120, China

**Keywords:** *Mycobacterium tuberculosis*, Rv3717, interleukin enhancer-binding factor 2, proteasomal degradation

## Abstract

*Mycobacterium tuberculosis* (*M. tuberculosis*) has developed some strategies to evade host immune responses through ubiquitination, thereby facilitating persistent mycobacterial infection. The Rv3717 protein has been identified as a peptidoglycan (PG) amidase that contributes to mycobacterial survival, but its exact mechanism is still unclear. The findings of this study indicate that Rv3717 inhibits mycobacterial clearance within pulmonary epithelial cells. To elucidate the molecular mechanisms by which Rv3717 facilitates persistent infection, we identified intracellular candidates interacting with Rv3717 using co-immunoprecipitation (Co-IP) combined with liquid chromatography–mass spectrometry (LC-MS/MS). The unique proteins are categorized into three functional networks: mRNA splicing, the immune system process, and the translation process through Protein–Protein Interaction (PPI) analysis. The candidate interacting proteins of Rv3717 are involved in interleukin-2 enhancer-binding factor 2 (ILF2) and TAF15, as well as the polyubiquitin chain (UBC) and E3 ubiquitin ligase TRIM21. Our results suggest that intracellular Rv3717 is likely to influence biological processes through the potential interacting proteins. Our findings confirmed that Rv3717 interacted with interleukin enhancer-binding factor 2 (ILF2) through Co-IP and immunofluorescence assays. Furthermore, Rv3717 was verified to bind with ubiquitin and be degraded through the proteasome system. More importantly, the ubiquitination of Rv3717 accelerated the proteasomal degradation of ILF2 and downregulated the expression of IL-2. This study is the first to propose that the ubiquitination of the mycobacterial membrane vesicle-associated protein Rv3717 facilitates the proteasomal degradation of ILF2, resulting in the downregulation of IL-2 expression. Overall, the role of intracellular Rv3717 in promoting mycobacterial survival is associated with its ubiquitination and the proteasomal degradation of ILF2.

## Simple Summary

*Mycobacterium tuberculosis* Rv3717 facilitates mycobacterial survival in host cells. This study is the first to verify that Rv3717 can be modified by polyubiquitin chains and promote ubiquitin-dependent proteasomal degradation of interleukin enhancer-binding factor 2 (ILF2), thus affecting the expression of IL-2, ILF2, P62, and LC3. More importantly, the changes can be recovered by Rv3717-K0 without ubiquitination. This suggests that ubiquitin-dependent proteasomal degradation induced by Rv3717 is likely to be associated with host cellular defense against *Mycobacterium tuberculosis*.

## 1. Introduction

*Mycobacterium tuberculosis* (*M. tuberculosis*) is an intracellular pathogen that is the causative agent of tuberculosis (TB). In 2023, there were an estimated 10.8 million new TB cases worldwide and 1.25 million deaths, indicating that TB remains a public health threat [1]. During *M. tuberculosis* infection, extracellular vesicles (EVs) containing mycobacterial components are released from infected macrophages and internalized into uninfected phagocytes to modulate host immunity, contributing to *M. tuberculosis* survival [2,3]. Thus, a crucial mechanism of mycobacterial persistence is related to immunomodulation of EVs.

The immunodominant components associated with EVs encompass glycolipids (lipoarabinomannan, LAM) and a diverse array of proteins, including lipoproteins, adhesins, and secretion-associated proteins [4]. Among these antigens, Rv3717 was identified as a membrane vesicle-associated protein through proteomic analysis [3]. Rv3717 consists of 241 amino acid residues, featuring an anchorless adhesin residing in the periplasm [5,6]. Functionally, Rv3717 has been identified as a peptidoglycan (PG) amidase, playing a crucial role in maintaining the integrity of the mycobacterial cell wall [7,8,9]. Furthermore, Rv3717 has been implicated in facilitating bacillary dissemination to the spleen and promoting intracellular survival in murine infection models [10,11,12]. However, the mechanism behind these observations is not clear.

**Figure 1:**
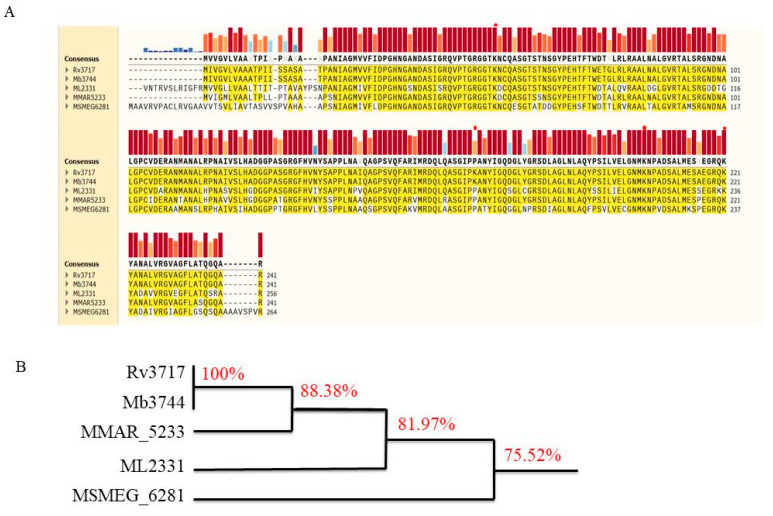
Multiple sequence alignments of Rv3717 and homologous proteins. (**A**) Comparison of amino acid sequences among Rv3717 and its homologous proteins. The consensus sequences are represented with red columns, and the height and color of other columns reflect the strength of conservation. Lys (K) residues are marked as a red/pink solid circle. (**B**) The evolutionary tree of Rv3717. Rv3717 is 100% identical with Mb_3744 of *M. bovis*, 88.38% identical with MMAR_5233 from *M. marinum*, 81.97% identical with ML_2331 of *M. leprae*, and 75.52% identical with MSMEG_6281 of *M. semgmatis*.

A study on membrane vesicles from *M. tuberculosis* demonstrated that they inhibit T-cell activation, and the inhibitory effect is attributed to the presence of LAM and unidentified components within MVs [2]. Intriguingly, *M. tuberculosis* lacking Rv3717 was able to be eliminated, but wild-type *M. tuberculosis* was found to remain viable during the chronic phase of infection [6]. The results demonstrated that Rv3717 contributed to mycobacterial persistence. The Rv3717 protein is characterized by high conservation in mycobacterium species (Figure 1). This structural conservation suggests that Rv3717 is essential for mycobacterial pathogenicity. Based on these observations, we propose a hypothesis that this phenomenon is likely associated with impaired adaptive immune responses.

Several studies have demonstrated that immunodominant antigens from *M. tuberculosis* act as decoys to induce T-cell exhaustion and anergy, thus weakening host immunity [13,14,15]. In general, antigens are presented on the cellular surface to modulate adaptive immune responses or interact with intracellular target proteins to manipulate host immunity [16]. Consequently, it is crucial to investigate Rv3717-interacting proteins and their effects on biological processes to improve understanding of the immunomodulatory mechanisms elicited by Rv3717.

## 2. Materials and Methods

### 2.1. Cells and Plasmid Construction

THP-1 was differentiated into macrophages (dTHP-1) using PMA (Sigma-Aldrich, St. Louis, MO, USA). dTHP-1 cells were cultured in RPMI-1640 medium with 10% fetal bovine serum (FBS), 1% penicillin/streptomycin (P/S), 0.05 mM β-Mercaptoethanol, and 20 ng/mL PMA. Human pulmonary alveolar epithelial cells (HPAEpiCs) were cultured in RPMI-1640 medium with 10% FBS and 1% P/S. A549 and HEK293T cells were grown in Dulbecco’s Modified Eagle Medium (DMEM, Gibco, Carlsbad, CA, USA) with 10% FBS and 1% P/S.

The genes were cloned via PCR amplification using the primers (F: 5′CGGAATTCATGATAGTCGGGGTACTC 3′; R: 5′ CGGGATCCCTAACGCGCCTGGCC 3′). After DNA sequencing, the fragments digested by restriction enzymes were ligated into vectors to construct recombinant plasmids. Rv3717 was cloned from *M. tuberculosis* genomic DNA into the pCS2_Flag vector, and then further ligated into the GV230 vector using HindIII and BamHI to construct GV230:: Flag-Rv3717. The Rv3717-K0 gene, coding 4 lysine mutations (K55R, K171R, K205R, and K221R), was synthesized by Sangon Biotech, Shanghai, China. After digestion with EcoRI and BamHI, it was inserted into the GV230_Flag vector to construct the GV230:: Flag-Rv3717-K0 plasmid.

The ILF2 gene was amplified using the primers (5′GGAATTCATGAGGGGTGACAGAGGC3′; 5′ GCTCTAGATCACTCCTGAGTTTCCATGC 3′) and cloned into the EcoRI and XbaI sites of the pCS2_HA vector to construct pCS2_HA::ILF2. The pCS2_HA::ILF2 recombinant vector was first digested by XbaI, and then blunt ends were formed using the Klenow fragment; GV230, digested by BamHI, was used to fill the blunt ends using Klenow fragment. The two plasmids were digested by HindIII and ligated to construct GV230::HA-ILF2.

### 2.2. The Detection of Mycobacterial Clearance Rate in Macrophages

The recombinant strains of *rM.smeg* and *rM.smeg*/Rv3717 were constructed using electroporation of wild-type *M. smegmatis* with pSUM_Rv3717-EGFP and pSUM_EGFP plasmids. The bacterial suspension was adjusted to an appropriate concentration based on colony-forming units (CFU). The suspension was repeatedly aspirated using a 26G needle syringe and dispersed for 20 min in an ultrasonic cleaner. THP-1 cells were inoculated into 96-well plates with three replicates and differentiated into dTHP-1 cells using 20 ng/mL PMA. Bacterial suspension was added to each well at a multiplicity of infection (MOI) of 1:100. The cells were incubated for 3 h with mycobacteria and treated using amikacin at a final concentration of 50 μg/mL for 1 h to eliminate extracellular bacteria, and then incubated for 0, 1, 3, and 6 h. The cells were broken using ddH_2_O at 37 °C for 20 min, and the released mycobacterial suspension was calculated using CFU/mL.

### 2.3. Co-Immunoprecipitation and LC–MS/MS

The candidate proteins interacting with Rv3717 were identified using co-immunoprecipitation (Co-IP) combined with liquid chromatography–mass spectrometry (LC–MS/MS). HPAEpiCs transfected with GV230::Flag_Rv3717 (HPAEpiC/Rv3717) and wild-type HPAEpiCs as a negative control (HPAEpiC/control) were collected. The whole-cell lysates from the two groups of cells were extracted, and the proteins were eluted on Anti-Flag M2 Affinity Gel beads (Sigma Aldrich, St. Louis, MO, USA). The extracts were separated using SDS–PAGE and the protein bands were subjected to LC–MS/MS analysis, which was carried out at PTM BIO (Hangzhou, China). The unique proteins of HPAEpiC/Rv3717 compared with the HPAEpiC/control were displayed. The unique proteins were analyzed through functional enrichment and the KEGG (Kyoto Encyclopedia of Genes and Genomes) pathway, and Protein–Protein Interaction network analysis was conducted using Protein–Protein Interaction (PPI) networks via the STRING database (https://string-db.org/), which was set at the highest confidence of 0.9 [17]. Additionally, the significant protein scores were set at greater than 70 in this study.

HEK293T cells were transiently co-transfected with GV230::Flag_Rv3717 and GV230::HA_ILF2 or GV230::HA_UBC using Lipo8000TM Transfection Reagent (Beyotime Bio, Shanghai, China). The medium was replaced after 12 h, and the cells were harvested after 36 h. The cells were washed with PBS and lysed with ice-cold lysis buffer for Co-IP (Wanlei Bio, Shenyang, China) with 1 mM PMSF. After 30 min on ice, the lysed cells were centrifuged at 12,000× *g* for 20 min at 4 °C to remove debris. Some of the soluble fraction was removed as input samples, while the remainder of the supernatant was incubated with anti-FLAG M2 Affinity Gel beads (Sigma Aldrich, St. Louis, MO, USA). All samples were gently rotated overnight at 4 °C, then washed with TBS five times. After removing the final washing buffer, the proteins were eluted from FLAG beads using 3× FLAG peptide (Beyotime Bio, Shanghai, China) for 2 h at 4 °C. The samples from the input and eluted proteins were separated using SDS–PAGE and evaluated via immunoblotting.

### 2.4. Immunofluorescence and Western Blotting

A549 cells were solely transfected or co-transfected with GV230::Flag_Rv3717 and GV230::HA_ILF2 for 48 h using Lipo8000^TM^ Transfection Reagent (Beyotime Bio, Shanghai, China). 293T cells were co-transfected with GV230::Flag_Rv3717 and GV230::HA_UBC for 48 h using Lipo8000^TM^ Transfection Reagent. The cells were washed with phosphate-buffered saline (PBS) containing 1% FBS and fixed with 4% paraformaldehyde for 15 min at room temperature. We added 0.2% Triton X-100 (Solarbio Life Sciences, Beijing, China) for 15 min at room temperature; then, the slides were washed and blocked with 3% BSA at 37 °C for 30 min. After washing, the slides were immunostained with specific antibodies of anti-FLAG-tag mouse mAb (Abmart Inc., Shanghai, China) and anti-HA-tag rabbit mAb (Abcam Inc., Cambridge, MA, USA). After washing, the slides were incubated with CoraLite 594-conjugated or CoraLite 488-conjugated IgG (Proteintech Group, Wuhan, China). The nucleus was stained with DAPI. Images of the localization were acquired via fluorescence microscopy and confocal laser scanning microscopy (Leica TCS SP5II, Leica Microsystems, Wetzlar, Germany).

To perform Western Blotting, the cells were collected at 24, 48, and 72 h after DNA transfection. The cells were placed in ice-cold RIPA lysis buffer (Wanlei Bio, Shenyang, China) with 1 mM PMSF; nuclear proteins and cytoplasmic proteins were isolated via a nuclear protein and cytoplasmic protein extraction kit (Wanlei Bio, Shenyang, China). The proteins were collected using centrifugation and quantified using BCA reagent (Wanlei Bio, Shenyang, China). Proteins were separated using 12% SDS–PAGE and evaluated via immunoblotting.

For immunoblotting, the proteins were first transferred onto polyvinylidene fluoride (PVDF) membranes (Cytiva, Dassel, Germany). The membranes were blocked with 5% non-fat milk in TBST buffer for 2 h at room temperature and then incubated with primary antibodies at 4 °C overnight, including anti-FLAG-tag mouse mAb (Abmart Inc., Shanghai, China), anti-HA-tag mouse mAb (Abmart Inc., Shanghai, China), as well as IL-2, followed by the incubation of HRP-conjugated secondary antibodies (Proteintech Group, Wuhan, China) at room temperature. We visualized the bands using ECL reagents (Tanon Science, Shanghai, China) and analyzed them using the Image-J 2.1.4.7 software (National Institutes of Health, Bethesda, MD, USA).

### 2.5. The Assessment of Proteasomal Degradation of Intracellular Proteins

To assess the effect of ubiquitination of Rv3717 on proteasome degradation, we transiently transfected HEK293T cells with the corresponding plasmids Gv230_Flag-Rv3717 and Gv230-Flag-Rv3717-K0 for 24 h, or co-transfected Gv230/Gv230_HA-ILF2 and Gv230_Flag-Rv3717/Gv230_HA-ILF2 for 24 h. The cells were treated with eukaryotic protein synthesis inhibitor cycloheximide (CHX, 20 μg/mL) and the proteasome inhibitor MG132 (10 μM) at the corresponding time points. The expression change in Rv3717, Rv3717-K0, and ILF2 was detected using Western Blotting.

## 3. Results

### 3.1. The Impact of Rv3717 on Mycobacterial Clearance and Immune Responses in Host Cells

**Figure 2:**
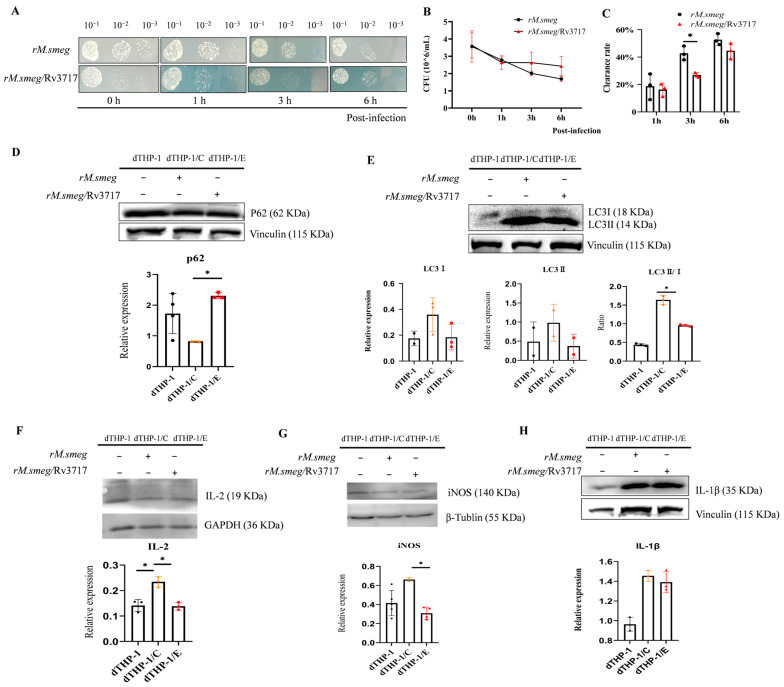
The impact of Rv3717 on mycobacterial clearance and immune responses in dTHP-1 cells. (**A**) Growth of intracellular *rM.smeg*/Rv3717 and *rM.smeg* (the control strain) on LB agar at 0, 1, 3, and 6 h post-infection; bacterial dilutions are shown on top. Images were taken after 48 h of incubation. (**B**) The colony-forming units (CFU) per milliliter (ml) of intracellular bacteria at 0, 1, 3, and 6 h post-infection are shown. (**C**) The graph shows the bacterial clearance rate in dTHP-1 cells during 1, 3, or 6 h, and the data was calculated according to CFU _(0 h–1/3/6 h)_/CFU _0 h_ × 100%. (**D**–**H**) The expression of autophagy-associated proteins (P62, LC3) and inflammatory factors (IL-2, IL-1β, iNOS) in dTHP-1 cells at 3 h post-infection was analyzed using Western Blotting. The dTHP-1 negative control group refers to differentiated THP-1 cells with PMA. dTHP-1/C represents dTHP-1 cells infected with the *rM.smeg* strain, and dTHP-1/E represents the dTHP-1 cells infected with the *rM.smeg*/Rv3717 strain. Whole-cell lysate was separated and transferred to PVDF membranes. The membranes were cropped and analyzed using respective antibodies, and all relevant lanes and reactive bands are displayed in the upper panels. The gray values of the target protein and the corresponding internal control bands are shown in the bottom panels. The black dots, pink triangles, and red squares, respectively, represent the dTHP-1, dTHP-1/C, and dTHP-1/E groups. (**D**) The expressions of P62 and the internal control Vinculin; (**E**) the expressions of LC3I, LC3II, and the internal control Vinculin, and the analysis of the LC3I/LC3II ratio; (**F**) the expressions of IL-2 and the internal control GAPDH; (**G**) the expressions of iNOS and the internal control β-Tublin; and (**H**) the expressions of IL-1β and the internal control Vinculin. Data information: All data are representative of at least three independent biological replicates, and error bars represent the mean ± standard deviation (SD) of triplicates. Unpaired Student’s *t*-test followed statistical analysis between two groups; an asterisk (*) indicates statistically significant (* *p* < 0.05).

We constructed a recombinant *M. smegmatis* strain with the pSUM_Rv3717-FGFP (*rM.smeg*/Rv3717) plasmid and its control strain (*rM.smeg*). The strains were used to infect dTHP-1 cells for 0, 1, 3, and 6 h, and the intracellular bacteria were released and cultured on agar plates (Figure 2A and Figure S1). The colony-forming units (CFU/mL) results showed a decline in intracellular bacterial load with the duration of post-infection. Notably, bacterial load in *rM.smeg*/Rv3717 group was greater than that observed in the *rM.smeg* negative control group (Figure 2B). By calculating bacterial clearance rate, we found that the *rM.smeg/Rv3717* strain exhibited a statistically lower clearance rate at 3 h post-infection in comparison with the control group (Figure 2C). Subsequently, Western Blotting was employed to detect the expression changes in the factors related to autophagy and inflammation at 3 h post-infection. In dTHP-1 cells, the *rM.semg*/Rv3717 strain infection resulted in a 2.80-fold increase in P62 expression (Figure 2D and Figure S2), while LC3II/I ratio (Figure 2E and Figure S3), IL-2 expression (Figure 2F and Figure S4) and iNOS expression (Figure 2G and Figure S5) decreased by 0.58-fold, 0.59-fold, and 0.47-fold, respectively, compared to the *rM.semg* infection group. However, a statistically significant change was not observed in the expression of IL-1β (Figure 2H and Figure S6). Collectively, these findings suggest that Rv3717 attenuates the clearance of intracellular bacteria and inhibits defense against bacteria in host cells.

### 3.2. Identification of Rv3717-Interacting Proteins

**Figure 3:**
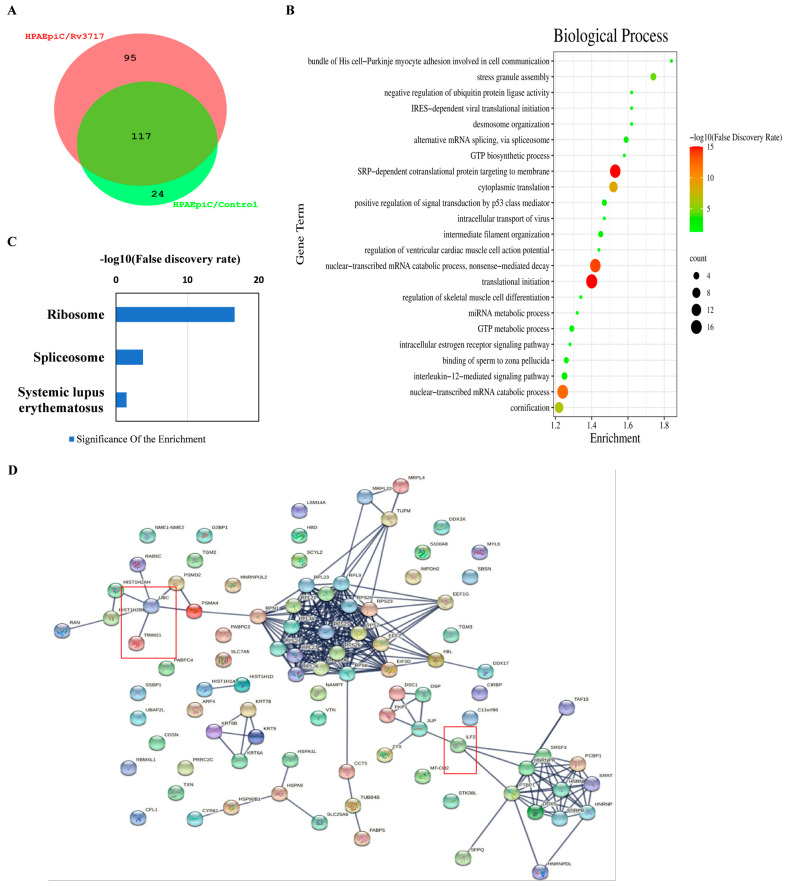
Functional analysis of the identified potential Rv3717-interacting proteins through Co-IP combining LC/MS-MS. (**A**) A Venn diagram of the proteins from the Rv3717-transfected group (HPAEpiC/Rv3717) and the negative control group (HPAEpiC/control) identified through LC-MS/MS analysis. The 95 unique proteins of the Rv3717-transfected group are indicated by a pink circle; the negative control group proteins are indicated by a green circle. (**B**) The 95 unique proteins were used to carry out the functional enrichment analysis, and biological process pathways with an enrichment effect strength exceeding 1.2 were visualized. The strength of the enrichment effect is >1.2. The false discovery rate indicates the statistical significance of the enrichments, and the diameter of the solid circle represents the number of annotated proteins. (**C**) Visualized KEGG enrichment pathways of the 95 unique proteins. The strength values of the enrichment effects of ribosome, spliceosome, and systemic lupus erythematosus pathways are 1.43, 1.05, and 0.95, respectively. The blue columns represent the false discovery rate, indicating the statistical significance of the enrichments. (**D**) Protein–Protein Interaction (PPI) networks of the 95 unique proteins were analyzed with the highest confidence of 0.9 via the STRING database; the red box indicates the Rv3717 interacting proteins mentioned in this study.

To clarify the molecular mechanism, we successfully identified 95 candidate proteins that interact with Rv3717 through Co-IP combined with LC-MS/MS (Figure 3A). The unique proteins with high hit scores (>70 protein score) are listed in Table 1. GO enrichment analysis indicated that biological processes with a strength score of over 1.2 were involved in myocyte adhesion, cell communication, alternative mRNA splicing, SRP-dependent co-translational protein targeting the membrane, and IL-12-mediated signaling pathways (Figure 3B). KEGG enrichment analysis of the 95 unique proteins indicated that ribosome, spliceosome, and systemic lupus erythematosus pathway displayed higher strength enrichment effects (Figure 3C). The unique proteins were categorized into three functional networks: mRNA splicing, the immune system process, and the translation process through Protein–Protein Interaction (PPI) analysis (Figure 3D). In the functional network cluster, we found that the domain associated with zinc fingers (DZF domain) is involved in interleukin-2 enhancer-binding factor 2 (ILF2, NF45) and TAF15; moreover, it was found that the polyubiquitin chain (UBC) and TRIM21 are related to ubiquitination, as illustrated in Figure 3D. Our results suggest that intracellular Rv3717 is likely to influence biological processes through the potential interacting proteins.

### 3.3. Rv3717-ILF2 and Rv3717-Ubiquitin Interactions

**Figure 4:**
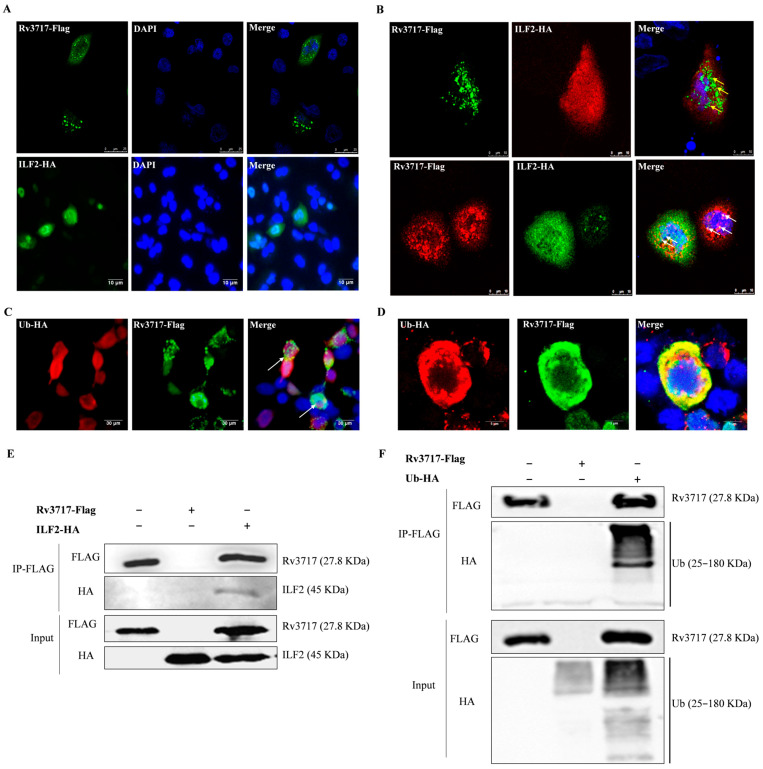
Interaction analysis of Rv3717 with ILF2 and ubiquitin. (**A**) A549 cells solely transfected by GV230::Flag_Rv3717 (the upper panels) or Gv230::HA_ILF2 (the bottom panels) for 48 h were analyzed via fluorescence microscope. The slides of the upper panels were stained with anti-FLAG-tag mouse mAb and incubated with CoraLite 488-conjugated goat anti-mouse IgG (Green); the slides of the bottom panels were stained with anti-HA-tag rabbit mAb and incubated with Green. The nucleus was stained with DAPI. (**B**) Confocal images of co-localization between Rv3717 and ILF2 (1200×). The cells were incubated using anti-FLAG-tag mouse mAb and anti-HA-tag rabbit mAb, followed by incubation with labeled IgGs. The upper panels show Rv3717 was stained with Green and ILF2 incubated with CoraLite 594-conjugated anti-rabbit IgG (Red); the bottom panels show that Rv3717 incubated with anti-mouse IgG (Red) and ILF2 incubated with anti-rabbit IgG (Green). The yellow arrows point to the yellow dots of co-location; the white arrows point to the white dots of co-location. (**C**) Co-transfected HEK293T cells with GV230::Flag_Rv3717 and GV230::HA_ubiquitin for 48 h were analyzed via fluorescence microscope. The cells were incubated using anti-FLAG-tag mouse mAb and anti-HA-tag rabbit mAb, followed by incubation with labeled IgGs. Rv3717 was stained with Green; ubiquitin was stained with Red. (**D**) Confocal images of co-localization between Rv3717 and ubiquitin (1200×). Rv3717 with Green; ubiquitin was stained with Red. (**E**,**F**) HEK293T cells were transfected with GV230::Flag_Rv3717 and/or Gv230::HA_ILF2 (**E**) and GV230::Flag_Rv3717 and/or Gv230::HA_Ub (**F**) for 48 h. The lysate part was removed as Input samples, and the remainder was incubated with anti-FLAG M2 Affinity Gel beads as IP-FLAG samples. The samples were analyzed using anti-FLAG rabbit mAb and anti-HA rabbit mAb using Western Blotting. The experiments were carried out with at least three independent biological replicates.

To determine the potential effects of Rv3717–ILF2 and Rv3717–ubiquitin interactions on biological processes, we observed the subcellular distribution of Rv3717 in host cells. The results showed that Rv3717_Flag was predominantly localized around the nuclear membrane, and ILF2_HA was identified in the cytoplasm and nucleus using the immunofluorescent assay (Figure 4A). After being co-transfected with GV230::Flag_Rv3717 and GV230::HA_ILF2, the co-localization of Rv3717 and ILF2 was confirmed via the observation of the overlapping dots, indicating distribution around the nuclear membrane (Figure 4B). Moreover, after HEK293T cells were co-transfected with GV230::Flag-Rv3717 and GV230::HA-ubiquitin, the co-localization of Rv3717 and ubiquitin was also confirmed using the immunofluorescent assay (Figure 4C) and confocal microscopy (Figure 4D). Co-IP results confirmed the co-precipitation of Rv3717 and ILF2 (Figure 4E and Figure S7), as well as Rv3717 and ubiquitin (Figure 4F and Figure S8). The findings confirm Rv3717–ILF2 and Rv3717–ubiquitin interactions, suggesting that Rv3717 may be modified by polyubiquitin chains and further affect the ILF2-associated pathways through its interaction, so as to exert the function of Rv3717.

### 3.4. The Ubiquitination of Rv3717 Promotes Proteasomal Degradation of ILF2

**Figure 5:**
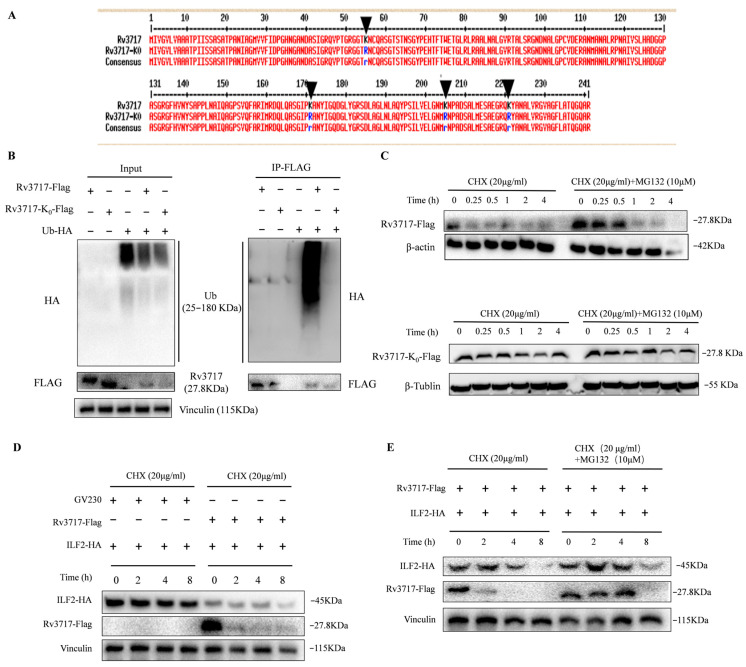
The ubiquitination of Rv3717 promotes proteasomal degradation of ILF2. (**A**) Comparison of amino acid sequences between Rv3717 and Rv3717-K0 (the lysine sites are indicated by black arrows) and amino acid mutations, with lysine (K) mutating to arginine (R). (**B**) HEK293T cells were co-transfected with GV230::Flag_Rv3717 or GV230::Flag_Rv3717-K0 and GV230::HA_Ub plasmids, whole-cell protein was extracted, and some of the samples were used as Input samples; the remaining samples were used as IP-FLAG samples. Input samples were identified using anti-FLAG rabbit mAb, anti-HA rabbit mAb, and anti-Vinculin mAb through Western Blotting. IP-FLAG samples were identified using anti-FLAG rabbit mAb and anti-HA rabbit mAb through Western Blotting. (**C**) After GV230::Flag_Rv3717/GV230::Flag_Rv3717-K0 plasmids were transfected into HEK293T cells for 24 h, the cells were treated with actinomycin (CHX) and/or MG132. The cells were collected at the indicated times to evaluate the levels of Rv3717-Flag and Rv3717-K0-Flag using anti-FLAG mAb through Western Blotting, and β-actin and β-tublin were used as internal controls. (**D**) HEK293T cells were co-transfected with GV230::Flag_Rv3717/GV230 and GV230::HA-_ILF2 for 24 h, and then treated with CHX for the indicated time. Whole-cell protein was extracted to identify the levels of ILF2 and Rv3717 using anti-ILF2 mAb and anti-FLAG mouse mAb through Western Blotting. (**E**) HEK293T cells were co-transfected with GV230::Flag_Rv3717 and GV230::HA_ILF2 for 24 h, and then treated with CHX and/or MG132 for the indicated time. Whole-cell protein was extracted to identify the levels of ILF2 and Rv3717 through Western Blotting. All experiments were carried out with at least three independent biological replicates.

To explore the effects of ubiquitination of Rv3717 on host immune responses, we constructed the GV230::Flag_Rv3717-K0 plasmid to express its mutation at K55R, K171R, K205R, and K221R (Figure 5A). The Co-IP result showed that Rv3717-K0 did not bind to ubiquitin (Figure 5B and Figure S9). To determine the effects of the ubiquitination of Rv3717 on proteasomal degradation, we transiently transfected HEK293T cells with plasmids GV230::Flag_Rv3717 and GV230::Flag_Rv3717-K0 for 24 h, and then treated them with cycloheximide (CHX) and proteasome inhibitor MG132 at different time points. The results showed that Rv3717 was degraded in a time-dependent manner and was almost completely degraded at 15 min, while Rv3717-K0 did not show degradation over time (Figure 5C and Figure S10). These results suggest that Rv3717 is degraded through the ubiquitin–proteasome pathway in host cells.

According to previous reports, ILF2 is degraded through ubiquitination, which affects the function and biological activity of the ILF2/ILF3 complex. Our findings indicate that ILF2 was degraded with CHX treatment. In the Rv3717 treatment group, the expression of ILF2 was significantly downregulated, and the protein degradation was obvious (Figure 5D and Figure S11). The degradation of ILF2 was suppressed after the addition of MG132, and the content of ILF2 at 2 h and 4 h was significantly higher than that at 0 h (Figure 5E and Figure S11). These results indicate that Rv3717 promotes the degradation of ILF2 through the ubiquitin–proteasome pathway.

### 3.5. Effects of Rv3717 Ubiquitination on the Expression of Intracellular Immunity-Related Factors

**Figure 6:**
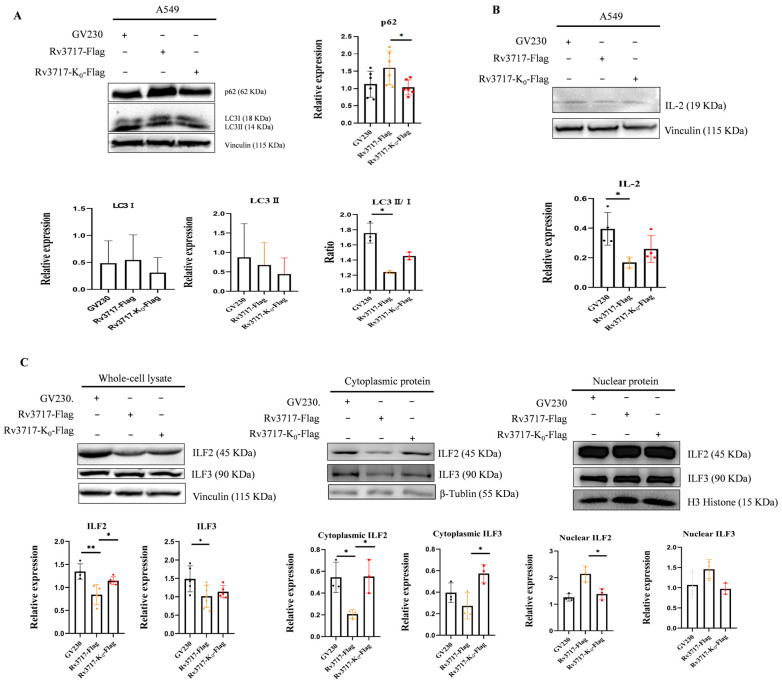
The effects of ubiquitination of Rv3717 on the expression of intracellular related factors. A549 cells were transfected with GV230 (the negative control group), GV230::Flag_Rv3717 (The Rv3717 group), and GV230::Flag_Rv3717-K0 (The Rv3717-K0 group), respectively, for 48 h. (**A**,**B**) Whole-cell proteins were extracted to detect the expression of proteins such as P62 and LC3I, LC3II and internal control Vinculin (**A**); IL-2 and internal control Vinculin (**B**). (**C**) The change expression of ILF2 and ILF3 was identified in whole-cell, the cytoplasm and the nucleus through Western Blotting. ILF2 and ILF3 were detected using anti-ILF2 mAb and anti-ILF3 mAb; Vinculin, β-Tublin, and H3 Histone were used as whole-cell, cytoplasmic, and nuclear protein internal controls, respectively. All data are representative of at least three independent biological replicates, and error bars represent mean ± SD. Unpaired Student’s *t*-test followed statistical analysis between two groups; asterisks (*^/^**) indicates statistically significant (* *p* < 0.05, ** *p* < 0.01).

We further employed A549 cells to evaluate the impact of Rv3717 ubiquitination on the expression of genes involved in cellular antibacterial defense mechanisms. P62 expression was increased by 1.42-fold and the LC3II/I ratio was reduced by 0.7-fold in the Rv3717 group compared to the negative control (NC) group. In contrast, the Rv3717-K0 group showed a 0.64-fold decrease in P62 expression and 1.17-fold increase in the LC3II/I ratio compared to the Rv3717 group (Figure 6A and Figure S12). The results suggest that Rv3717 boosts P62 accumulation and hinders LC3 activation in A549 cells, which may be reversed by the ubiquitination-deficient Rv3717-K0.

It was observed that IL-2 level decreased by 0.42-fold in the Rv3717 group compared to the NC group, and increased by 1.54-fold in the Rv3717-K0 group compared to the Rv3717 group (Figure 6B and Figure S13). The result indicates that host cells significantly inhibited the production of IL-2, which is associated with ubiquitination.

Furthermore, our data revealed that ILF2 significantly decreased by 0.63-fold and 0.38-fold in whole-cell and cytoplasmic protein, respectively, and increased by 1.7-fold in nuclear protein in the Rv3717 group compared to NC group. Conversely, ILF2 increased by 1.36-fold and 2.67-fold in whole-cell and cytoplasmic protein, and decreased by 0.64-fold in nuclear protein in the Rv3717-K0 group compared to the Rv3717 group. ILF3 follows a similar pattern; Rv3717 decreased ILF3 by 0.68-fold and 0.69-fold in whole-cell and cytoplasmic protein and increased it by 1.36-fold in nuclear protein, whereas Rv3717-k0 resulted in a 1.12-fold and 2.1-fold increase in ILF3 expression in whole-cell and cytoplasmic protein and decreased it by 0.67-fold in nuclear protein (Figure 6C and Figure S14).

The findings suggest that the immunosuppression effects induced by Rv3717 are associated with its ubiquitination. Inhibiting the ubiquitination of Rv3717 appears to modulate cellular autophagy, specifically interacting with ILF2 and influencing IL-2 secretion, which may impact T-cell immunity.

## 4. Discussion

Mycobacterium species release antigens based on extracellular vesicles to manipulate the host immune system. Previous reports have demonstrated that Rv3717, as a membrane vesicle-associated protein, facilitated intracellular survival during the later stages of murine infection models and enhanced the survival of *M. smegmatis* through the inhibition of host innate immune and Caspase-dependent apoptosis [10]. Here, the non-pathogenic *M. smegmatis* strain, which possesses a similar cell wall structure to *M. tuberculosis*, was employed to assess intracellular survival. Our results indicated that the ability of macrophages to eliminate *rM.smeg/Rv3717* was diminished compared with the control strain. This suggests that Rv3717 enhances mycobacterial survival within macrophages, thereby promoting the persistence of intracellular *M. smegmatis*.

Ubiquitination is a post-translational modification characterized by the covalent attachment of either a single ubiquitin moiety or polyubiquitin chain (UBC) to the lysine residue of a substrate protein. Each ubiquitin molecule contains seven lysine residues (K6, K11, K27, K29, K33, K48, and K63) and a free N-terminus methionine residue (M1), facilitating the formation of a variety of ubiquitin linkages that result in structurally diverse signals. Pathogenic bacteria secrete various effector proteins that target and regulate other effectors within host cells, thereby facilitating bacterial survival and proliferation. During pathogen invasion, host cells activate the ubiquitination system to exert a “marking–alerting–clearing” effect, which in turn activates autophagy, NF-κB signaling pathways, inflammasomes, and other mechanisms to eliminate intracellular pathogens. Nevertheless, pathogenic bacteria have evolved diverse strategies to counteract these host defense systems. For instance, the effector protein LubX from *Legionella* pneumophila mediates the proteasomal degradation of another effector, SidH, while also promoting the degradation of Cdc2-like kinase 1 to modulate host cellular immunity [18,19]. *M. tuberculosis* has exploited ubiquitin-dependent processes in host cells, which is one of the crucial mechanisms for its immune evasion and pathogenicity [20]. *M. tuberculosis* secreted effector protein Rv0222 has been confirmed to suppress host immunity through K11 ubiquitin modification based on E3 ubiquitin ligase ANAPC2 [21]. *M. tuberculosis* tyrosine phosphatase PtpA binds ubiquitin to inhibit NF-κB pathway activation and pro-inflammatory cytokine production [22]. These studies strongly demonstrate that the selective elimination of invading mycobacteria requires the association of ubiquitin with intracellular virulence factors.

This is the first study to demonstrate that intracellular Rv3717 can bind ubiquitin and be degraded through the proteasomal pathway. It has been reported that *M. tuberculosis* infection enhances ubiquitin-dependent proteasomal degradation of HERC2 associated with nuclear receptor coactivator 4 (NCOA4) as a cargo receptor in ferritin degradation through TRIM21, resulting in suppression of host ferritin metabolism to facilitate *M. tuberculosis* intracellular survival [23]. This suggests that *M. tuberculosis* likely exploit the ubiquitin-dependent proteasomal degradation system to facilitate the immune evasion.

The finding that Rv3717 facilitated intracellular mycobacterial survival in macrophages prompted us to investigate whether and how Rv3717 facilitates bacterial survival. This study demonstrated that *M. smegmatis* strain can activate autophagy in macrophages, characterized by LC3 activation and P62 reduction. In contrast, *rM.smeg*/Rv3717 strain disrupts this process, causing P62 accumulation and inhibition of LC3 activation. These findings demonstrate that Rv3717 likely hinders autophagolysosome maturation induced by *M. smegmatis*. Interestingly, the ubiquitination-deficient Rv3717-K0 led to statistically significant decrease in P62 levels and an increase in LC3 activation. The results suggest that the ubiquitination of Rv3717 is associated with the inhibition of phagolysosome maturation, thereby promoting the persistence and survival of *M. tuberculosis* within host cells.

Additionally, we demonstrated that the ubiquitination of Rv3717 accelerated proteasomal degradation of ILF2, reduced the level of ILF2 protein in the cytoplasm, and inhibited IL-2 expression. ILF2 was initially identified as a subunit of the nuclear factor of activated T cells (NFAT) and is an important transcription factor complex for IL-2 expression in T cells [24,25]. ILF2 usually forms heterodimers with ILF3 to regulate gene expression and T-cell activation [26]. Our findings demonstrate that Rv3717 reduced the levels of ILF2 and ILF3 in the cytoplasm and did not affect their content in the cell nucleus, suggesting that Rv3717 might promote the ubiquitin-dependent degradation of ILF2 through the interaction with ILF2 in the cytoplasm, modulating IL-2 expression.

More importantly, the results including the autophagy inhibition, ILF2 stability and IL-2 expression were recovered in the Rv3717-K0 group, indicating that inhibiting the ubiquitination of Rv3717 contributes to the recovery of defense against mycobacteria. Therefore, it is crucial to elucidate the E3 ubiquitin ligases responsible for ILF2 and Rv3717. Our findings indicate that TRIM21 is one of the identified candidate E3 ubiquitin ligases that interact with Rv3717 through Co-IP combined with LC-MS/MS. In future studies, we aim to verify the effects of TRIM21 knockdown on ILF2 degradation and T-cell activation in macrophages.

## 5. Conclusions

This study is the first to demonstrate that *M. tuberculosis* membrane vesicle-associated protein Rv3717 can be modified by polyubiquitin chains, influencing ubiquitin-dependent proteasomal degradation of ILF2 in macrophages. The associated E3 ubiquitin ligase can be considered a potential target for host-directed therapies aimed at inhibiting the intracellular persistence of *M. tuberculosis*. Future investigations will aim to elucidate the specific E3 ubiquitin ligases, deubiquitinases, and the types of ubiquitination related to Rv3717.

## Figures and Tables

**Table 1:** The specific interacting proteins of Rv3717 with high scores listed according to LC–MS/MS analysis.

Accession	Protein Names	Gene Names	MW [kDa]	Protein Score	Sequence Coverage (%)	Unique Peptides	Peptides	# PSMs
P15924	Desmoplakin	DSP	331.57	389.59	3.45	8	8	8
Q15942	Zyxin	ZYX	61.24	150.67	11.71	4	4	4
P26599	Polypyrimidine tract-binding protein 1	PTBP1	57.19	149.23	8.1	2	2	2
P52597	Heterogeneous nuclear ribonucleoprotein F	HNRNPF	45.64	142.03	8.19	2	2	3
P14923	Junction plakoglobin	JUP	81.69	139.51	4.43	3	3	3
P17844	Probable ATP-dependent RNA helicase DDX5	DDX5	69.1	138.77	6.03	2	3	3
P23246	Splicing factor, proline- and glutamine-rich	SFPQ	76.1	135.79	3.82	2	2	2
Q08554	Desmocollin-1	DSC1	99.92	111.94	2.24	1	1	1
Q9BXP5	Serrate RNA effector molecule homolog	SRRT	100.6	94.49	4.45	3	3	3
P51991	Heterogeneous nuclear ribonucleoprotein A3	HNRNPA3	39.57	79.1	6.61	2	2	2
O14979	Heterogeneous nuclear ribonucleoprotein D-like	HNRNPDL	46.41	76.05	3.81	1	1	1
Q92804	TATA-binding protein-associated factor 2N	TAF15	61.79	71.83	4.05	1	2	2
Q12905	Interleukin enhancer-binding factor 2	ILF2	43.04	70.35	3.59	1	1	1

# PSMs (Peptide-Spectrum Matches) represent the number of matches between MS/MS and the theoretical peptide sequences.

## Data Availability

The data presented in this study are available on request from corresponding author Tao Jiang. The data are not publicly available due to the strict management of our team.

## Supplementary Materials

The following supporting information can be downloaded at: https://www.mdpi.com/article/10.3390/biology14101414/s1, Figure S1: Images of bacterial colonies growing on agar plates. Figures S2–S14: Western Blotting Figures source; Figure S15: Fluorescence microscope and Confocal images source.
