# Supplementary material for: The Ubiquitination of Mycobacterium tuberculosis Rv3717 Promotes Proteasomal Degradation of Interleukin Enhancer-Binding Factor
Source: Biology (Basel). 2025 Oct 14;14(10):1414. doi: 10.3390/biology14101414 (PMC12561918; doi:10.3390/biology14101414)
Supplement: Supplementary file 1 [file biology-14-01414-s001.zip › biology-3832217-supplementary.pdf]

Supplementary Materials

The ubiquitination of *Mycobacterium tuberculosis* Rv3717 promotes proteasomal degradation of interleukin enhancer-binding factor

Figure 2-A source

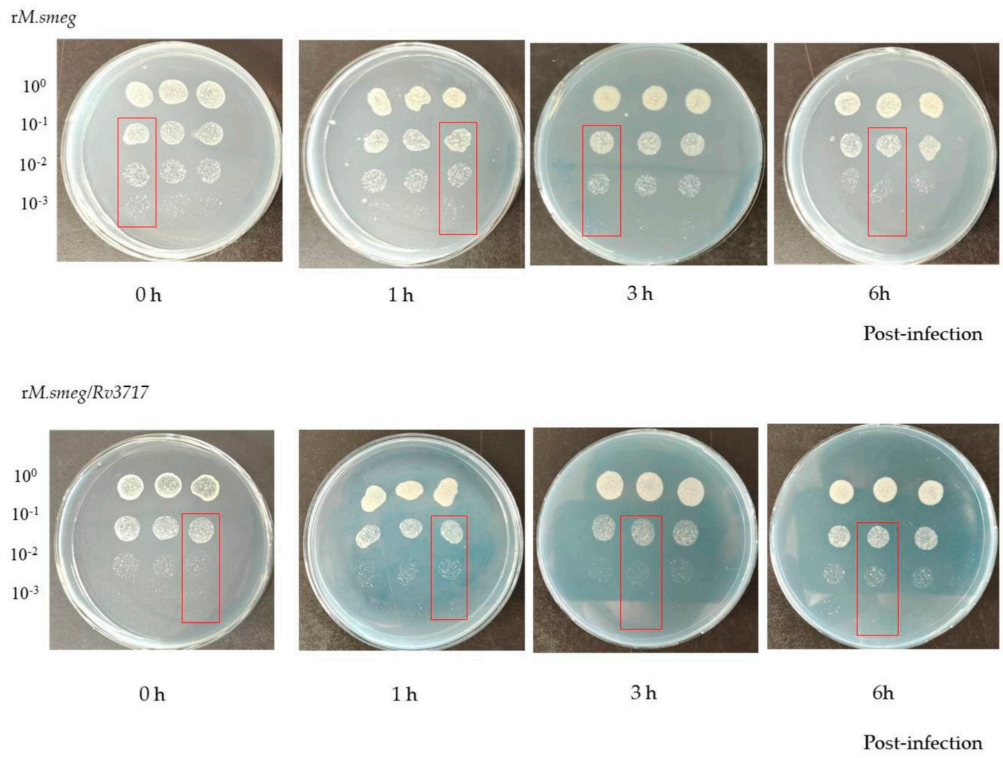

Figure S1: Images of bacterial colonies growing on agar plates. dTHP-1 cells were infected with r*M.smeg*/Rv3717 and r*M.smeg* at an multiplicity of infection (MOI) of 100 for 3 h. The cells were treated with 50 μg/ml amikacin for 1 h. The time point was considered as 0 h post-infection. The cells were cultured at with 100 μl of RPMI-1640 medium containing 10% FBS. Cells were lysed at 0, 1, 3, and 6 h post-infection. Intracellular bacteria were diluted in a gradient of 1:10; 1:10^2; 1:10^3 and 1:10^4, and 10 μl was dropped onto LB agar plates for cell counting. Agar plates were taken after 48 h of incubation and each plate represents three biological replicates.

Figure 2-D source

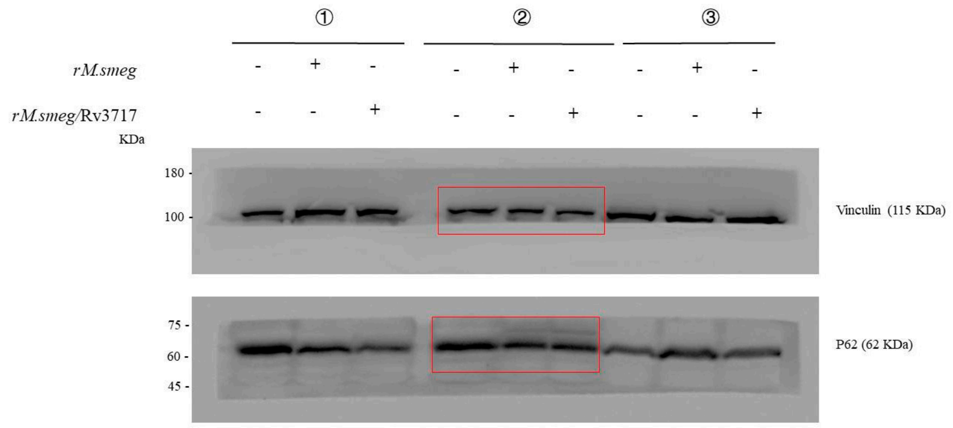

Figure S2: Western Blotting membrane of P62 (~ 62KDa) and the internal control Vinculin (~ 115 KDa). The total proteins of 40 μg were transfected onto polyvinylidene fluoride (PVDF) membranes (Cytiva, Dassel, Germany) using the electrotransfer apparatus (LIUYI Biotechnology Co., LTD., Beijing, China). The membranes were cropped at red bands of 75 KDa (Mw.) of 3-color Prestained Protein Standards (AG11919, Accrate Biology, Changsha, China). The one with < 75 KDa was detected Anti-P62 polyclonal rabbit antibody (Proteintech Group, 18420-1-AP，1:1000，Wuhan, China), and another was detected with anti-Vincullin antibody (Sigma Aldrich, V9131, 1:10000，St. Louis, MO, USA). The membranes were followed by the incubation of HRP-conjugated secondary antibodies (Proteintech Group, Wuhan, China) at room temperature. The bands were visualized using ECL reagents (Tian Neng Biotechnology Co., LTD, Shanghai, China). The images were converted to grayscale and analyzed with Image J 2.1.4.7 (National Institutes of Health, Bethesda, MD, USA). The analysis procedure of Image J 2.1.4.7 in this study is followed: Launch the Image J software, select File→Open in the menu bar, find the target file and open it. 1. Convert to grayscale image: In the menu bar, select Image→Type→8-bit to convert the image to grayscale. 2. Remove the Background: Select Process→Subtract Background to remove the influence of the background. The default Rolling ball radius is 50. 3. Set measurement parameters: Select Analyze→Set Measurements, check the result items to be analyzed, and then click OK. 4. Measure the gray scale value: Use the rectangle or ellipse tool to select the area to be measured, and then use the shortcut key Ctrl + M or the menu command "Analyze→Measure" to measure.

Figure 2-E source

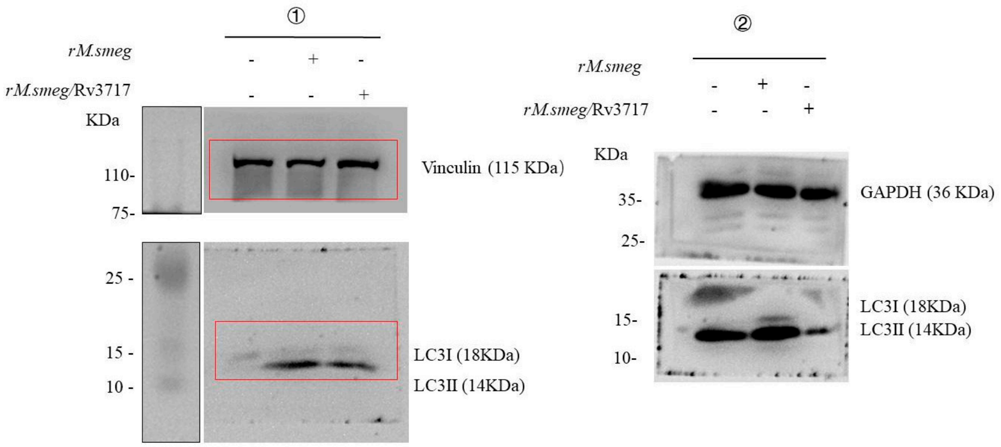

Figure S3: Western Blotting membranes of LC3Ⅰ (~ 18KDa), LC3Ⅱ(~ 14kDa) and the internal control Vinculin (~ 115 KDa) or GAPDH. The total proteins of 40 μg were transfected onto polyvinylidene fluoride (PVDF) membranes using the electrotransfer apparatus (LIUYI Biotechnology Co., LTD., Beijing, China). The membranes were cropped at red bands of 75 KDa (Mw.) of 3-color Prestained Protein Standards (AG11919, Accrate Biology, Changsha, China). The one with < 75 KDa was detected Anti-LC3 polyclonal rabbit antibody (Proteintech Group, 14600-1-AP，1:1000，Wuhan, China), and another was detected with anti-Vincullin Antibody (Sigma Aldrich, V9131, 1:10000，St. Louis, MO, USA). The membranes were followed by the incubation of HRP-conjugated secondary antibodies (Proteintech Group, Wuhan, China) at room temperature. The bands were visualized using ECL reagents (Tian Neng Biotechnology Co., LTD, Shanghai, China). The images were analyzed with Image J 2.1.4.7.

Figure 2-F source

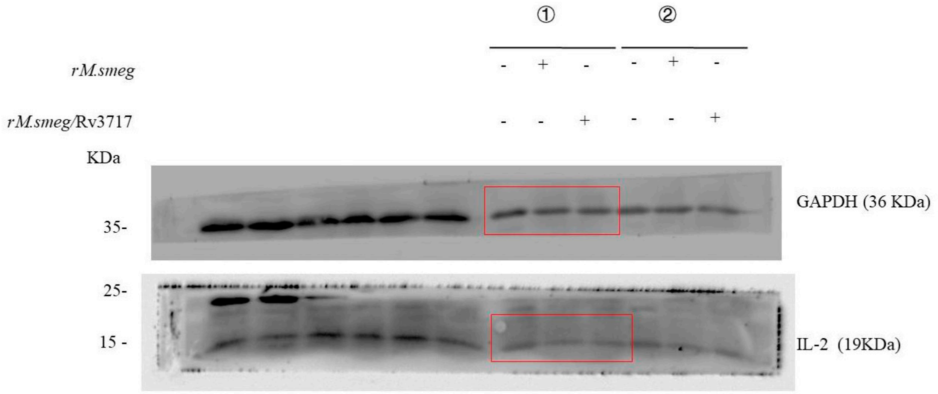

Figure S4: Western Blotting membranes of IL-2 (~19 KDa) and the internal control GAPDH (~ 36 KDa) for Western Blotting. The total proteins of 40 μg were transfected onto polyvinylidene fluoride (PVDF) membranes using the electrotransfer apparatus. The membranes were cropped at red bands of 45 KDa (Mw.) of 3-color Prestained Protein Standards (AG11919, Accrate Biology, Changsha, China). The one with < 35 KDa was detected Anti-IL-2 polyclonal rabbit antibody (Proteintech Group, 26156-1-AP，1:1000，Wuhan, China) and another was detected with anti-GAPDH polyclonal rabbit antibody (Bioword Technology, Inc, AP0066，1:10000, Nanjing, China). The membranes were followed by the incubation of HRP-conjugated secondary antibodies (Proteintech Group, Wuhan, China) at room temperature. Blot images were visualized using ECL reagents (Tian Neng Biotechnology Co., LTD, Shanghai, China). The images were analyzed with Image J 2.1.4.7.

Figure 2-G source

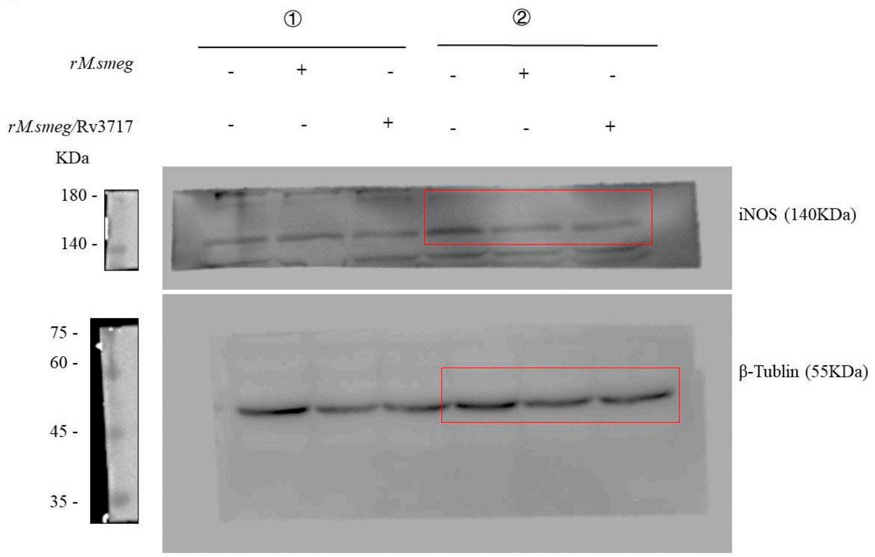

1.

Figure S5: Western Blotting membranes of iNOS (~140 KDa) and the internal control β-Tublin (~ 55 KDa). The total proteins of 40 μg were transfected onto polyvinylidene fluoride (PVDF) membranes using the electrotransfer apparatus (LIUYI Biotechnology Co., LTD., Beijing ,China). The membranes were cropped at red bands of 140 KDa (Mw.) of 3-color Prestained Protein Standards (AG11919, Accrate Biology, Changsha, China). The one with < 75 KDa was detected Anti-iNOS polyclonal rabbit antibody (Wanlei, WL0992a，1:1000，Shenyang, China), and another was detected with anti-β-Tublin polyclonal rabbit antibody (Proteintech Group, 80713-1-RR，1:1000，Wuhan, China). The membranes were followed by the incubation of HRP-conjugated secondary antibodies (Proteintech, USA) at room temperature. Blot images were visualized using ECL reagents (Tian Neng Biotechnology Co., LTD, Shanghai, China). The images were analyzed with Image J 2.1.4.7.

Figure 2-H source

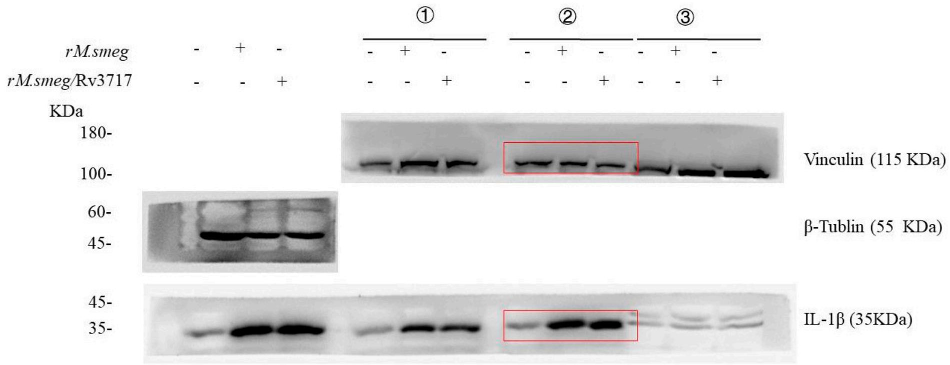

Figure S6: Western Blotting membrane of IL-1β (~35 KDa) and the internal control β-Tublin (~ 55 KDa)/ Vinculin (~ 115 KDa). The total proteins of 40 μg were transfected onto PVDF membranes using the electrotransfer apparatus (LIUYI Biotechnology Co., LTD., Beijing, China). The membranes were cropped at red bands of 100 KDa (Mw.) of 3-color Protein Standards (AG11919, Accrate Biology, Changsha, China). The one with < 45 KDa was detected Anti-IL-1β polyclonal rabbit antibody (Proteintech Group, 26048-1-AP, 1:1000, Wuhan, China), and another was detected with anti-β-Tublin polyclonal rabbit antibody (Proteintech Group, 80713-1-RR, 1:1000, Wuhan, China)/anti-Vincullin antibody (Sigma Aldrich, V9131, 1:10000，St. Louis, MO, USA). The membranes were followed by the incubation of HRP-conjugated secondary antibodies (Proteintech Group, Wuhan, China) at room temperature. Blot images were visualized using ECL reagents (Tian Neng Biotechnology Co., LTD, Shanghai, China). The images were analyzed with Image J 2.1.4.7.

Figure 4-E source

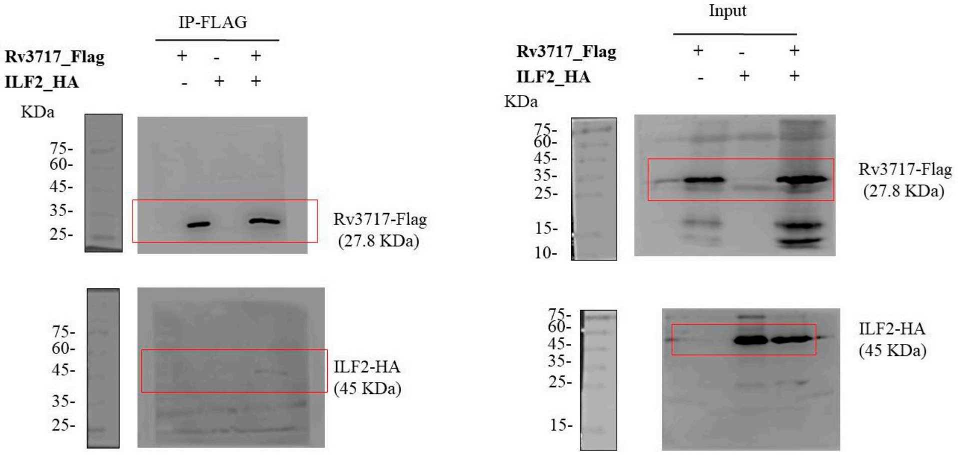

Figure S7: Western Blotting membrane of Rv3717-Flag (27.8 KDa) and ILF2-HA (45 KDa). The total proteins (Input: 40 μg, IP: 30μL of processed IP protein lysate) were transfected onto PVDF membrane using the electrotransfer apparatus (LIUYI Biotechnology, China). The membranes of Rv3717-Flag were detected Anti-FLAG-Tag Mouse mAb (Abmart, M20008, 1:5000, Shanghai, China), and the membranes of ILF2-HA were detected with HA-Tag(26D11) Mouse Antibody (Abmart, M20003，1:1000, Shanghai, China). The membranes were followed by the incubation of HRP-conjugated secondary antibodies (Proteintech Group, 1:5000, Wuhan, China) at room temperature. Blot images were visualized using ECL reagents (Tian Neng Biotechnology Co., LTD, Shanghai, China). The images were analyzed with Image J 2.1.4.7.

Figure 4-F source

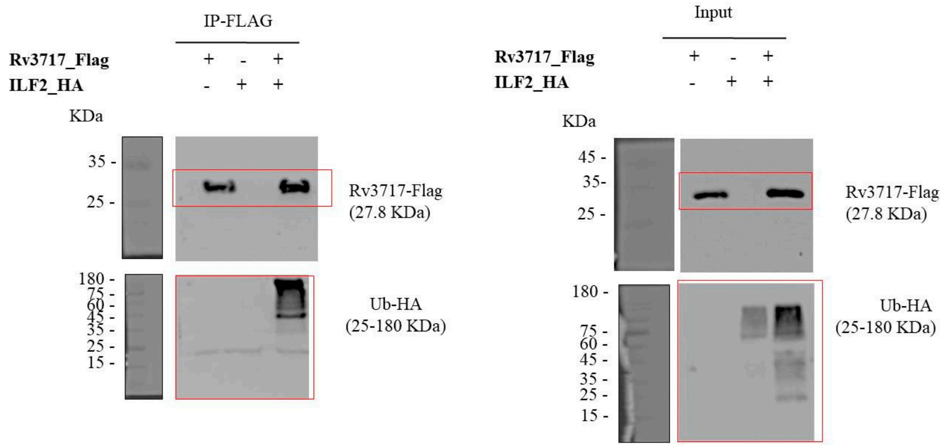

Figure S8: Western Blotting membrane of Rv3717-Flag (27.8 KDa) and Ub-HA (25-180 KDa). The total proteins (Input: 50 μg, IP: 30μL of processed IP protein lysate) were transfected onto PVDF membranes using the electrotransfer apparatus (LIUYI Biotechnology, Beijing, China). The membrane pieces of Rv3717-Flag were detected Anti-Flag-Tag Mouse mAb (Abmart, M20008, 1:5000, Shanghai, China), and the membranes of Ub-HA were detected with HA-Tag(26D11) Mouse Antibody (Abmart, M20003, 1:1000, Shanghai, China). The membranes were followed by the incubation of HRP-conjugated secondary antibodies (Proteintech Group, 1:5000, Wuhan, China) at room temperature. Blot images were visualized using ECL reagents (Tian Neng Biotechnology Co., LTD, Shanghai, China). The images were analyzed with Image J 2.1.4.7.

Figure 5-B source

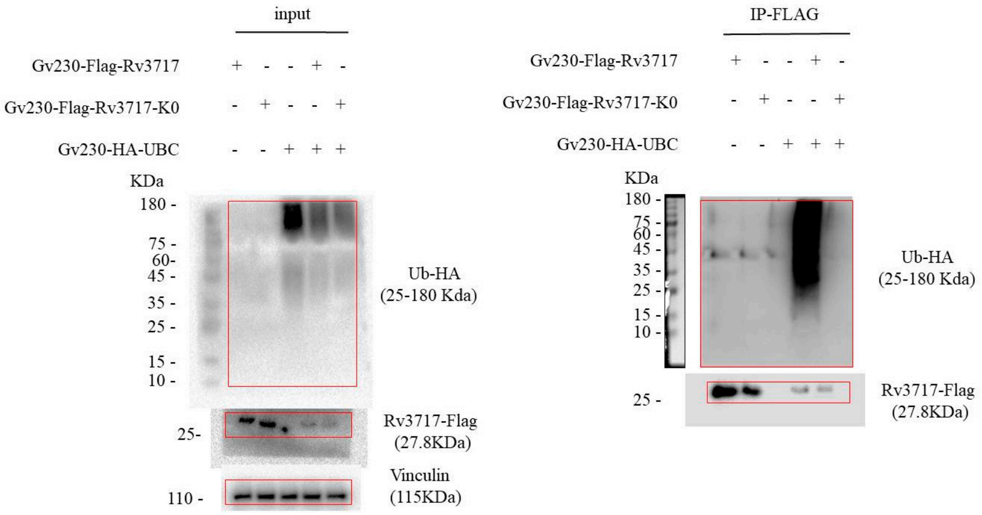

Figure S9: Western Blotting membrane of Ub-HA (25~180 KDa)/ Rv3717-Flag (27.8 KDa) and the internal control Vinculin (~ 115 KDa). The total proteins of 40 μg were transfected onto PVDF membranes using the electrotransfer apparatus (LIUYI Biotechnology Co., LTD., Beijing, China). The membrane was detected Anti-HA polyclonal Mouse antibody (Abmart, M20003，1:1000，Shanghai, China)/ Anti-Flag polyclonal Mouse antibody (Abmart, M20008L，1:5000，Shanghai, China), and another was detected with anti-Vincullin Antibody (Sigma Aldrich, V9131, 1:10000，St. Louis, MO, USA). The membranes were followed by the incubation of HRP-conjugated secondary antibodies (Proteintech Group, 1:5000, Wuhan, China) at room temperature. Blot images were visualized using ECL reagents (Tian Neng Biotechnology Co., LTD, Shanghai, China). The images were analyzed with Image J 2.1.4.7.

Figure 5-C source

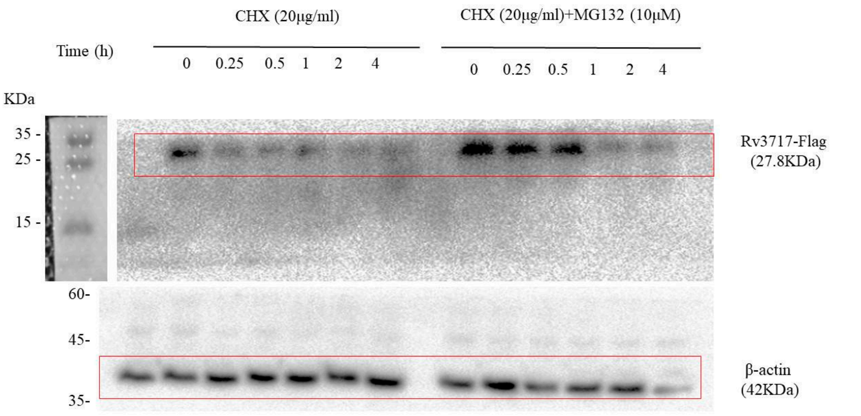

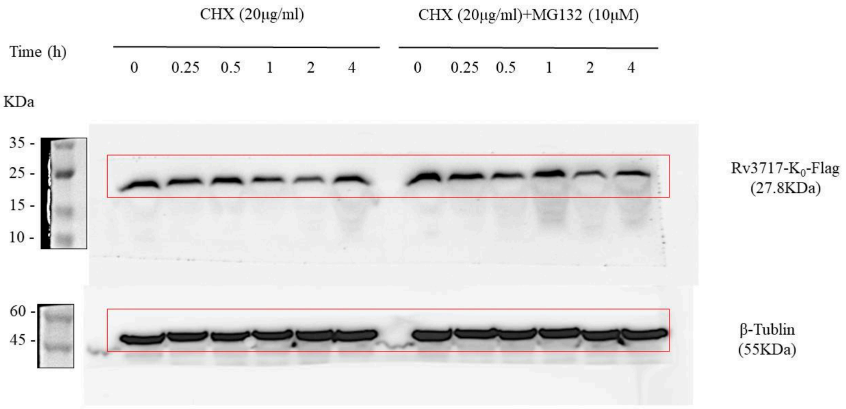

Figure S10: Western Blotting membranes of Rv3717-Flag (~27.8 KDa) and the internal control β-actin (~ 42 KDa); Rv3717-K0-Flag (~27.8 KDa) and the internal control β-Tublin (~ 55 KDa). The total proteins of 40 μg were transfected onto PVDF membranes using the electrotransfer apparatus (LIUYI Biotechnology Co., LTD., Beijing). The membranes were cropped at red bands of 15-35 KDa (Mw.) of 3-color Prestained Protein Standards (AG11919, Accrate Biology, Changsha, China). The one with 35-60 KDa was detected Anti-Flag polyclonal Mouse antibody (Abmart, M20008L，1:5000，Shanghai, China), and another was detected with anti-β-actin polyclonal rabbit antibody (Servicebio, GB15003，1:1000，Wuhan, China). The membranes were followed by the incubation of HRP-conjugated secondary antibodies (Proteintech Group, 1:5000, Wuhan, China) at room temperature. Blot images were visualized using ECL reagents (Tian Neng Biotechnology Co., LTD, Shanghai, China). The images were analyzed with Image J 2.1.4.7.

Figure 5 –D source

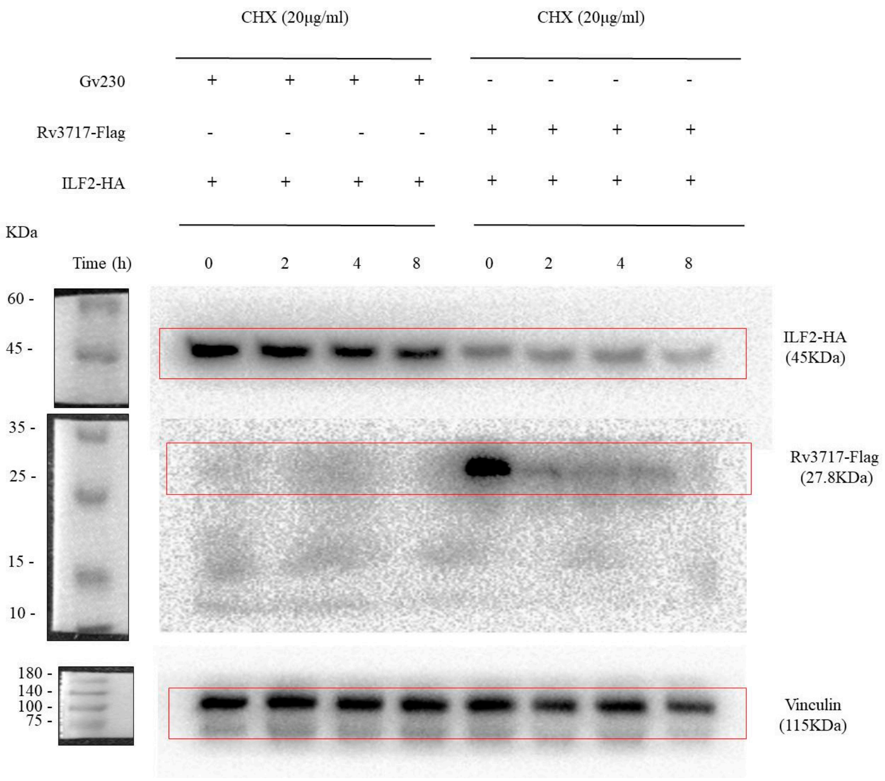

Figure 5-E source

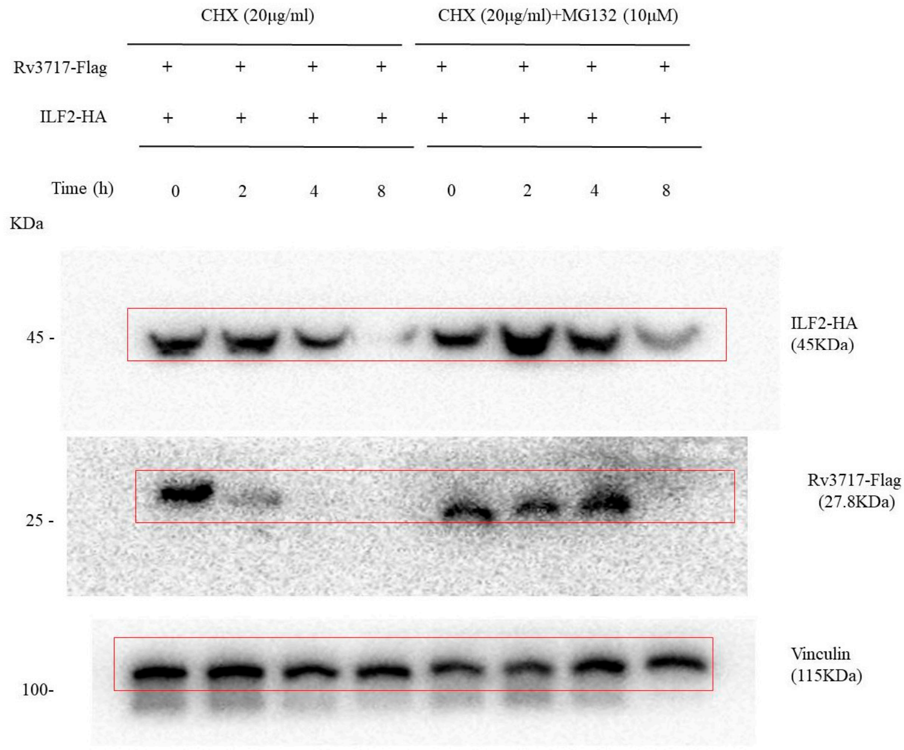

Figure S11: Western Blotting membrane of Rv3717-Flag (~27.8 KDa) /ILF2-HA (~45 KDa) and the internal control Vinculin (~ 115 KDa). The total proteins of 40 μg were transfected onto PVDF membranes using the electrotransfer apparatus (LIUYI Biotechnology Co., LTD., Beijing, China). The membranes were cropped at red bands of 100 KDa (Mw.) of 3-color Prestained Protein Standards (AG11919, Accrate Biology, Changsha, China). The one with 35-60 KDa was detected Anti-HA polyclonal Rabbit antibody (Servicebio, GB151252，1:1000，Wuhan, China) and the one with 25-35 KDa was detected Anti-Flag polyclonal Mouse antibody (Abmart, M20008L，1:5000，Shanghai, China) and another was detected with anti-Vincullin Antibody (Sigma Aldrich, V9131, 1:10000，St. Louis, MO, USA). The membranes were followed by the incubation of HRP-conjugated secondary antibodies (Proteintech Group, 1:5000, Wuhan, China) at room temperature. Blot images were visualized using ECL reagents (Tian Neng Biotechnology Co., LTD, Shanghai, China). The images were analyzed with Image J 2.1.4.7.

Figure 6 -A source

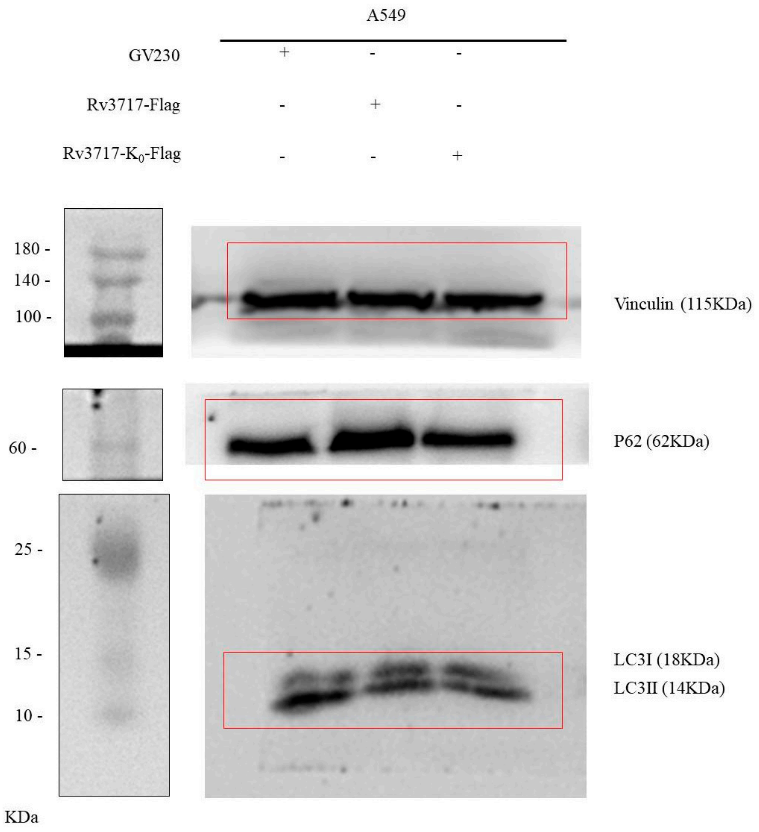

Figure S12: Western Blotting membrane of P62 (~62 KDa) /LC3 (14~18 KDa) and the internal control Vinculin (~ 115 KDa). The total proteins of 40 μg were transfected onto PVDF membranes using the electrotransfer apparatus (LIUYI Biotechnology Co., LTD., Beijing, China). The membranes were cropped at red bands of 100 KDa (Mw.) of 3-color Prestained Protein Standards (AG11919, Accrate Biology, Changsha, China). The one with 35-75 KDa was detected Anti-P62 polyclonal rabbit antibody (Proteintech Group, 18420-1-AP, 1:1000，Wuhan, China) and the one with 10-35 KDa was detected Anti-LC3 polyclonal rabbit antibody (Proteintech Group, 14600-1-AP, 1:1000, Wuhan, China) and another was detected with anti-Vincullin Antibody (Sigma Aldrich, V9131, 1:10000，St. Louis, MO, USA). The membranes were followed by the incubation of HRP-conjugated secondary antibodies (Proteintech Group, 1:5000, Wuhan, China) at room temperature. Blot images were visualized using ECL reagents (Tian Neng Biotechnology Co., LTD, Shanghai, China). The images were analyzed with Image J 2.1.4.7.

Figure 6 –B source

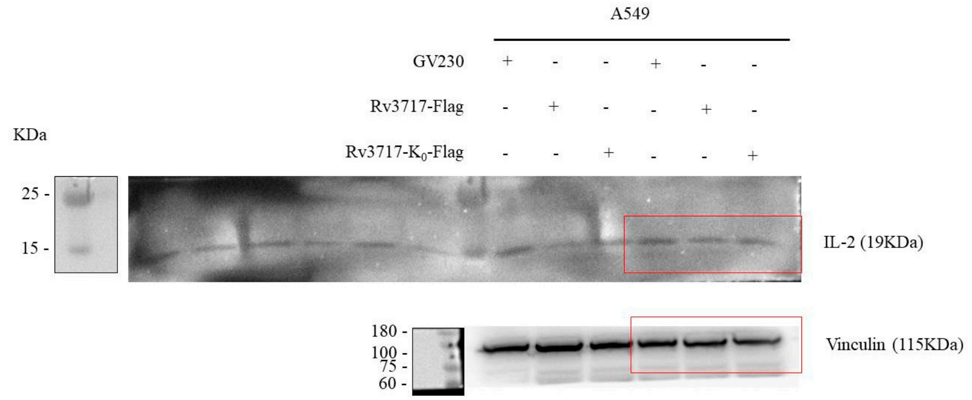

Figure S13: Western Blotting membrane of IL-2 (~19 KDa) and the internal control Vinculin (~ 115 KDa). The total proteins of 40 μg were transfected onto PVD mem, China). The membranes were cropped at red bands of 60 KDa (Mw.) of 3-color Prestained Protein Standards (AG11919, Accrate Biology, Changsha, China). The one with 15-25 KDa was detected Anti-IL-2 polyclonal rabbit antibody (Proteintech Group, 26156-1-A, 1:1000, Wuhan, China) and another was detected with anti-Vincullin Antibody (Sigma Aldrich, V9131, 1:10000，St. Louis, MO, USA). The membranes were followed by the incubation of HRP-conjugated secondary antibodies (Proteintech Group, 1:5000, Wuhan, China) at room temperature. Blot images were visualized using ECL reagents (Tian Neng Biotechnology Co., LTD, Shanghai, China). The images were analyzed with Image J 2.1.4.7.

Figure 6-C source

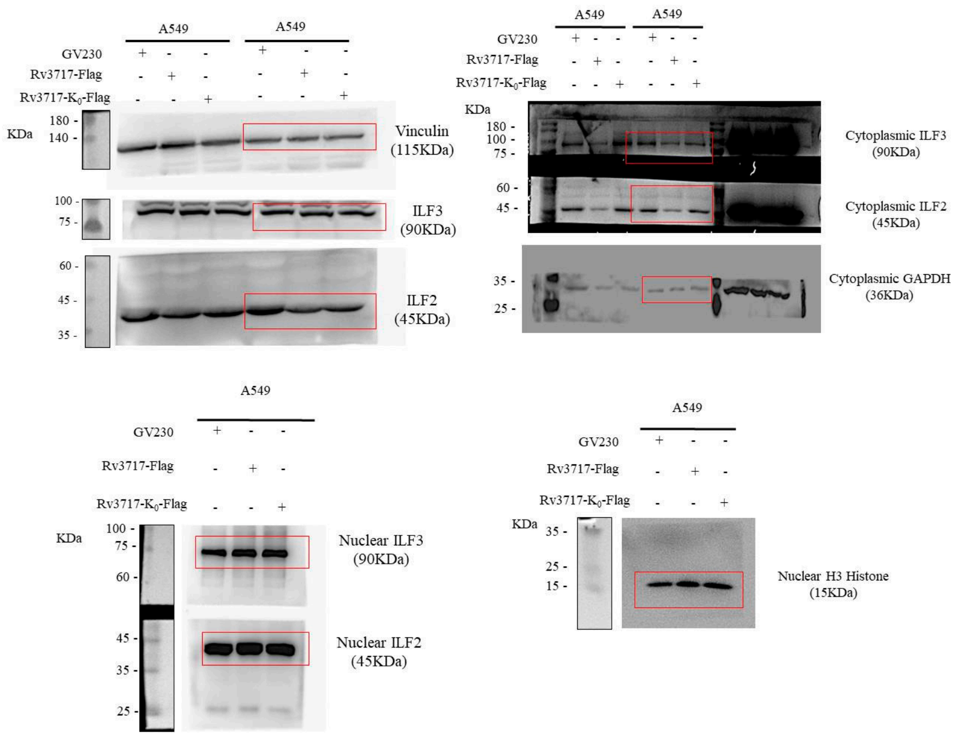

Figure S14: Western Blotting membrane of ILF2 (~45 KDa)/ILF3 (~90 KDa) and the internal control Vinculin (~ 115 KDa). The total proteins of 40 μg were transfected onto PVDF membranes using the electrotransfer apparatus (LIUYI Biotechnology Co., LTD., Beijing, China). The membranes were cropped at red bands of 100 KDa (Mw.) of 3-color Prestained Protein Standards (AG11919, Accrate Biology, Changsha, China). The one with 60-100 KDa was detected Anti-ILF3 polyclonal rabbit antibody (Proteintech Group, 19887-1-AP, 1:1000, Wuhan, China) and one with 35-60 KDa was detected Anti-ILF2 polyclonal rabbit antibody (Proteintech Group, 14714-1-AP, 1:1000, Wuhan, China) and another was detected with anti-Vincullin Antibody (Sigma Aldrich, V9131, 1:10000，St. Louis, MO, USA) and another was detected with anti-GAPDH polyclonal rabbit antibody (Bioword, AP0066，1:10000，Nanjing, China)and another was detected with anti-H3 Histone polyclonal Mouse antibody (Proteintech Group, 68345-1-lg, 1:10000, Wuhan, China). The membranes were followed by the incubation of HRP-conjugated secondary antibodies (Proteintech Group, 1:5000, Wuhan, China) at room temperature. Blot images were visualized using ECL reagents (Tian Neng Biotechnology Co., LTD, Shanghai, China). The images were analyzed with Image J 2.1.4.7.

Figure 4-A source

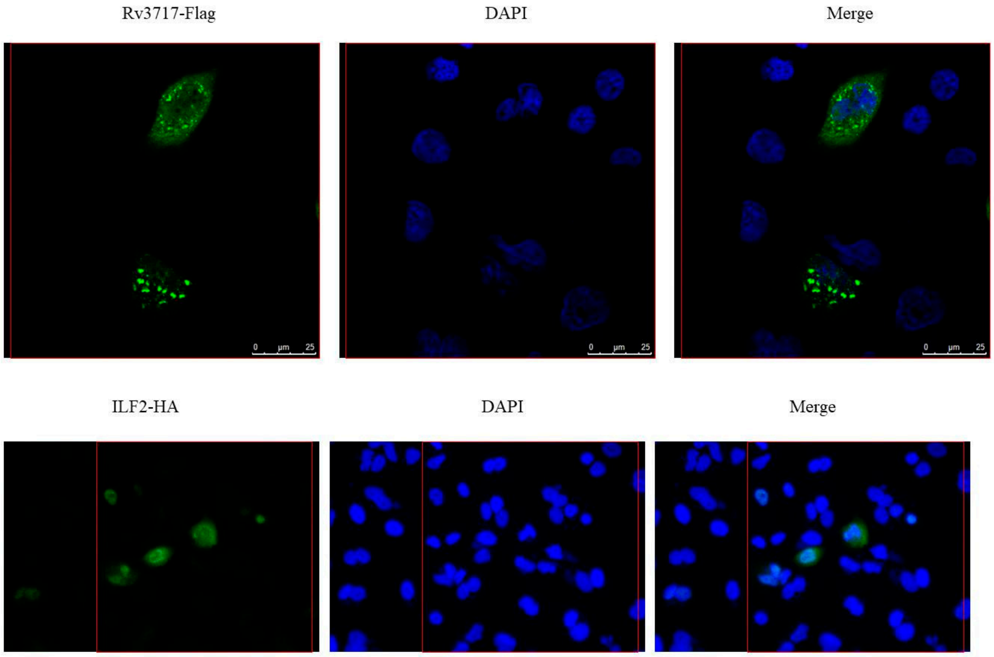

Figure 4-B source

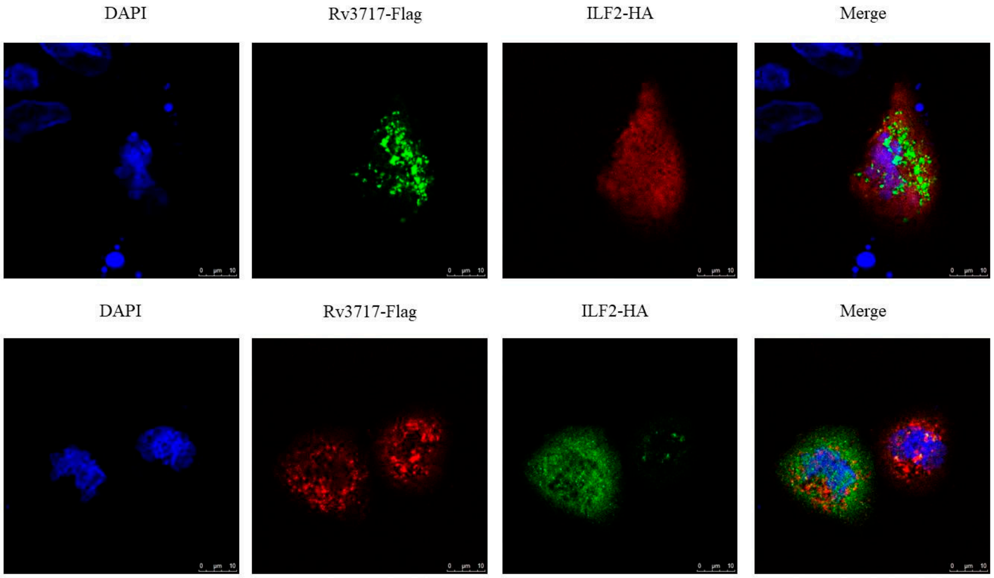

Figure 4-C source

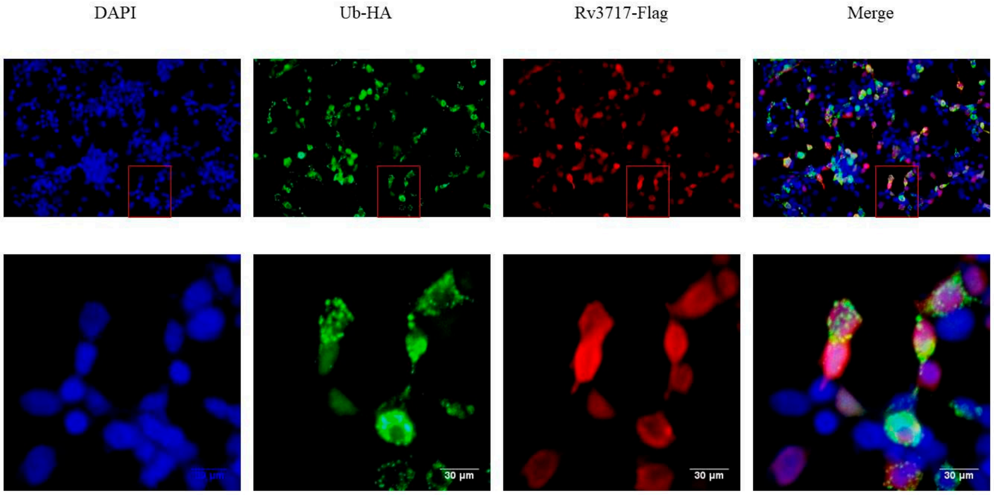

Figure 4-D source

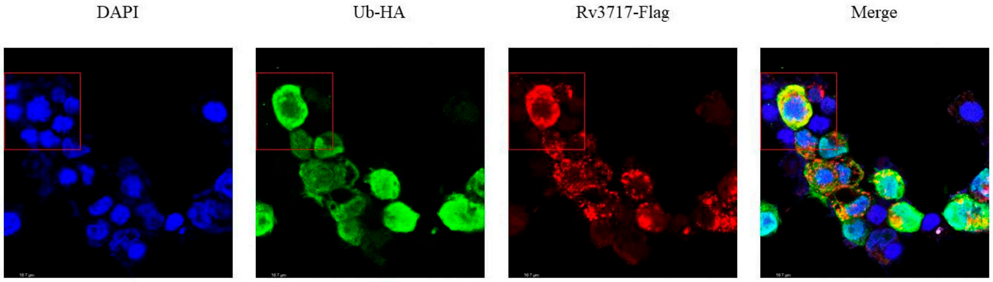

Figure S15: Fluorescence microscope and Confocal images source. (A) A549 cells solely transfected by GV230::Flag_Rv3717 (the upper panels) or Gv230::HA_ILF2 (the bottom panels) for 48 h were analyzed via fluorescence microscope. The slides of the upper panels were stained with Anti-Flag-tag mouse mAb (Abmart Inc, Shanghai, China) and incubated with CoraLite 488-conjugated goat anti-mouse IgG (Proteintech Group, Proteintech Group, 14714-1-AP, 1:1000, Wuhan, China); the slides of the bottom panels were stained with anti-HA-tag rabbit mAb (Abcam Inc, Cambridge, MA, USA, UK) and incubated with CoraLite 488-conjugated goat anti-rabbit IgG (Proteintech Group, Proteintech Group, SA00013-2-AP, 1:1000, Wuhan, China. The nucleus was stained with DAPI.
(B) Confocal images of co-localization between Rv3717 and ILF2 (1200×). The cells were incubated using Anti-Flag-tag mouse mAb (Abmart Inc, Shanghai, China) and anti-HA-tag rabbit mAb (Abcam Inc, Cambridge, MA, USA, UK), followed by incubation with labeled IgGs. The upper panels showed that Rv3717 was stained with CoraLite 488-conjugated goat anti-mouse IgG (Proteintech Group, Proteintech Group, 14714-1-AP, 1:1000, Wuhan, China). ILF2 was stained using CoraLite

594-conjugated goat anti-rabbit IgG (Proteintech Group, Proteintech Group, SA00013-4 -AP, 1:1000, Wuhan, China); the bottom panels represent that Rv3717 was stained with 594-conjugated goat anti-mouse IgG (Proteintech Group, SA00013-3-AP, 1:1000, Wuhan, China) and ILF2 was stained by CoraLite 488-conjugated goat anti-rabbit IgG (Proteintech Group, Proteintech Group, SA00013-2-AP, 1:1000, Wuhan, China); yellow arrows point to the yellow dots of co-location and white arrows point to white dots of co-location.
